# Assessing the clinical utility of pre-operative neutrophil–lymphocyte ratio as a predictor of clinicopathological parameters in patients being treated for primary breast cancer

**DOI:** 10.1007/s10549-025-07615-8

**Published:** 2025-01-20

**Authors:** Burce Isik, Matthew G. Davey, Alisha A. Jaffer, Juliette Buckley, James A. L. Brown, Chwanrow Baban, Bridget Anne Merrigan, Shona Tormey

**Affiliations:** 1 University of Limerick School of Medicine, Limerick, Ireland; 2 Symptomatic Breast Unit, University Hospital Limerick, Limerick, Ireland; 3 Department of Biological Sciences, University of Limerick, Galway, Ireland; 4 Limerick Digital Cancer Research Centre (LDCRC), University of Limerick, Limerick, Ireland

**Keywords:** Breast cancer, Immunology, Oncological outcomes, Precision oncology, Personalised medicine

## Abstract

**Background:**

There is a paucity of data supporting the role of neutrophil**–**lymphocyte ratios (NLR) to determine clinicopathological parameters in patients being treated for primary breast cancer.

**Aims:**

To evaluate the association between pre-operative NLR and clinicopathological parameters in patients diagnosed with breast cancer.

**Methods:**

A retrospective cohort study was performed. This included consecutive patients indicated to undergo surgery for primary breast cancer at University Hospital Limerick between January 2010 and June 2017. NLR was expressed as a continuous variable. Univariable and multivariable linear regression analyses were used to determine the correlation between NLR and clinicopathological data. Data analytics was performed using SPSS v29.0.

**Results:**

673 patients met the inclusion criteria. Overall, the median preoperative NLR is 2.63 (standard deviation: 1.42). At univariable analysis, patient age (beta coefficient: 0.009, 95% confidence interval (CI) 0.001–0.017, P = 0.027), tumour size (beta coefficient: 0.013, 95% CI 0.005–0.021, P = 0.001), and human epidermal growth factor receptor-2 status (beta coefficient: − 0.370, 95% CI − 0.676–0.065, P = 0.017) were all predicted using NLR. However, at multivariable analysis, tumour size was the sole parameter predictable by NLR (beta coefficient: 0.011, 95% CI 0.002–0.019, P = 0.013).

**Conclusions:**

This study demonstrates that pre-operative NLR may serve as an independent predictor of tumour size in patients being treated with primary breast cancer. Ratification of these preliminary findings is warranted before robustly adopted into clinical practice.

## Introduction

Breast cancer is a heterogeneous disease with an increasing incidence in the Western world [1]. Fortunately, the molecular classification of the disease has facilitated personalised multimodal treatment strategies, which have translated to enhanced outcomes for the majority of those diagnosed with the disease [2]. It is important to note that there may be important biochemical information that is routinely available from basic patient workup, which may prove useful in predicting certain aggressive phenotypical characteristics of such tumours [3]. Therefore, the identification of new low-cost diagnostic biomarkers which aid diagnosis is important, and their relevance in the context of treatment and prognosis of breast cancer may also be of importance [4–6]. Thus, translational research efforts have focused on providing such biomarkers which may aid contemporary breast cancer diagnosis [3].

Evaluating the impact of the tumour microenvironment upon various epithelial cancer subtypes has been the objective of oncological research for several years now [7]. Inflammation is a well-established hallmark of cancer, which has propagated investigation to assess the clinical role of inflammatory markers [8], such as neutrophils and lymphocytes, in both the tumour microenvironment and circulation of those who succumb to breast cancer diagnoses [9]. Neutrophil**–**lymphocyte ratio (NLR) is a calculation of the total neutrophil count divided by the total lymphocyte count, which seems to serve as a biomarker which expresses the balance of anti-tumour and tumour-promoting effects of circulating cells, thus, offering potential value as a predictive biomarker into oncogenesis and development [8, 9].

At present, there are preliminary data supporting the prognostic use of NLR in breast cancer for predicting oncological outcomes such as response to neoadjuvant chemotherapy, and survival outcomes such as recurrence-free (RFS) and overall survival (OS) outcomes [10–12]. Furthermore, there are emerging data which suggests that NLR may provide data with utility in serving in deciphering breast cancer molecular subtype [13]. In spite of these promising findings, there remains a paucity of studies which evaluate the value of using NLR to potentially predict routine clinicopathological characteristics of those treated for primary breast cancer. In particular, such a biomarker may be useful in less well-resourced hospitals in healthcare economies challenged by less access to diagnostics. Accordingly, the current study aimed to evaluate the clinical utility of pre-operative NLR as a predictor of clinicopathological parameters in patients with primary breast cancer.

## Methods

Local hospital ethical approval was sought and obtained. A single-centre, retrospective observational cohort study was undertaken, including consecutive patients undergoing primary breast cancer surgery at an Irish tertiary referral centre (University Hospital Limerick (UHL)), with an associated academic institution. (University of Limerick (UL)). Review of a prospectively maintained institutional database for patients treated in this unit was performed, with data augmented through verification of clinic letters, mammogram reports, and blood work values. The study was performed and reported by the Strengthening the Reporting of Observational Studies in Epidemiology (STROBE) guidelines for observation studies [14].

### Patient selection

The inclusion criteria for the current study included adult female patients, aged 18 years or older, who were diagnosed and treated for a new diagnosis of breast cancer between January 1st, 2010, and June 1st, 2017 in UHL. All cancer diagnoses were confirmed by core biopsy or radiologically suspicious lesion concerning for malignancy discussed in a multidisciplinary meeting. Those who underwent primary surgery during that period were included in the analysis. Excluded from this cohort were patients who received neoadjuvant chemotherapy, had metastatic (M1) disease at presentation, patients that declined surgical intervention, as well as patients who presented with recurrent disease either from outside of the time period or during the same time period, so as not to duplicate numbers. Patients who underwent prophylactic mastectomy which yielded histology positive for invasive carcinoma were excluded from the analysis. Participants without complete circulatory biomarker values were excluded from the analysis.

### Patient follow-up

Patient follow-up was conducted in person until five years post-operatively before being discharged from the service. This was achieved using clinic letters and imaging results prior to June 1, 2022. Hospital computers were used to further delineate long-term follow-up. The last formal review of medical notes was performed in November 2023.

### Triple assessment

Patients presented for triple assessment in the specialised breast cancer tertiary referral centre: a consultant breast surgeon performed clinical breast examinations on presentation, tissue biopsies were analysed by a consultant pathologist with expertise in breast pathology, and radiological assessment was conducted by a specialist breast consultant radiologist by mammography and/or ultrasound scanning. Tumour staging was performed in accordance with the American Joint Committee on Cancer (AJCC), version 8 Guidelines [15].

### Histopathologic assessment and immunohistochemistry

Oestrogen (ER) and progesterone (PR) receptor status on tumour specimens were analysed using the 2010 American Society of Clinical Oncology/College of American Pathologists (ASCO/CAP) histopathological consensus guidelines, although reporting was performed using the Allred scoring system [16, 17]. Human epidermal growth factor receptor-2 (HER2/neu) status was determined using immunohistochemistry, and patients scoring 2 + proceeded for fluorescence in situ hybridization to confirm HER2 status. Each specimen underwent histopathological grading in accordance with the Elston-Ellis modification of the Scarff-Bloom-Richardson grading system (as per the World Health Organisation Classification of Tumours Guidelines) [18]. Tumour lymphatic invasion was evaluated utilising IHC staining with D2-40 [19]. Vascular invasion was assessed by IHC using CD34 [20]. Perineural invasion was determined using IHC staining with S-100 and a broad-spectrum keratin stain (AE1/AE3) [21]. Ki-69 was evaluated using MIB1 antibody testing [22].

### Neutrophil–lymphocyte ratio

In the 30 days prior to cancer resection, peripheral venesection was performed as a component of formal pre-operative assessment. Thereafter, neutrophil count and lymphocyte count were recorded, and neutrophil**–**lymphocyte ratios (NLR) were calculated using the following formula:

$${\text{NLR}}\, = \,absolute \, neutrophil \, count/absolute \, lymphocyte \, count.$$

### Statistical analysis

Continuous variables are reported as medians with standard deviation (SD), while categorical variables are reported as frequencies and percentages. The numerical data were asssumed to be parametric. Linear regression results are reported as beta coefficient with a 95% confidence interval (CI) to decipher the relationship and directionality of clinicopathological data and NLR. All tests of significance were 2 tailed, with *P* < 0.050 indicating statistical significance. Descriptive analysis and regression analysis were conducted with SPSS (Version 29.0). Clinicopathological patient data were analysed using descriptive statistics. Fisher’s exact (¶), Chi-squared (χ2), one-way analysis of variance (ANOVA, □), and Kruskal Wallis (§) tests were used as appropriate. Survival outcomes and patterns of metastasis were also recorded.

## Results

### Clinicopathological information

In total, 673 patients met the inclusion criteria. The mean age at diagnosis was 57.96 ± 13.79 years (range 26–88). Two hundred and thirteen patients were pre-menopausal, while 17 were peri-menopausal, and 328 were post-menopausal. The majority of patients (79.2%) have been diagnosed with invasive ductal carcinoma (IDC), followed by invasive lobular carcinoma (ILC) (12.5%) and ductal carcinoma in situ (DCIS) (4.9%), exclusively. One hundred and eighty-nine patients had associated DCIS while 54 did not. Most patients’ tumour was staged T2 (45.2%), followed by T1 (38.2%) while only 7.4% and 1.2% of the patients were T3 and T4, respectively. Forty-three patients had Tis/T0 breast cancer (BC). More than half of the patients (57.9%) had grade 2 BC while 167 had grade 3 and 59 had grade 1 BC. Four hundred and two patients had N-stage 0 along with 167 N-stage 1, 59 N-stage 2, and 34 N-stage 3. Most patients (628) had unilateral BC while 31 had bilateral BC. One hundred and fifty-two patients had no lymphatic invasion while 61 had invasion with the remaining patients’ status unknown. The majority of patients have hormone receptor-positive BC; 516 ER + and 432 PR + . Five hundred and fifty-three patients have HER2− and 106 have HER2 + BC. The mean tumour size was 25.71 ± 16.191 mm.

### Management strategies

Three hundred and forty-five patients had breast-conserving surgery while 326 patients underwent mastectomy. Four hundred and twenty-seven patients had either sentinel lymph node biopsy (SLNB) or sentinel lymph node dissection (SLND) while 235 patients either had axillary lymph node biopsy (ALNB) or axillary lymph node dissection (ALND). Adjuvant chemotherapy was received by 289 patients (42.9%), while 70% received adjuvant radiation therapy, and 75.8% received adjuvant endocrine therapy. Eighty patients received extended endocrine therapy.

### Oncological and survival outcomes

Eighty-two per cent (552) of the patients had no recurrence while 622 patients had no local recurrence. Local recurrence at 5 years has been observed in 47 patients. Overall, 91 patients had metastatic BC with the most common site of metastasis being the bone (35) followed by multiple sites (19), lung (11), lymph nodes (8), liver (6), and brain (4). The patients were followed up after the operation for 66.85 ± 27.98 months. The time to death is calculated by subtracting the date of surgery from the date of death and ranges from 0 to 10 years. One hundred and twenty-four patients of the total cohort were deceased within 10 years, while 99 patients died within 5 years after surgery. The mean time to death of 124 deceased patients was 3.61 ± 2.37 years. The descriptive statistics of the patients with primary breast cancer are shown in Table 1.

**Table 1:** Descriptive statistics of the patient population

Variables		
Age at diagnosis	Mean ± SD (range); median	57.96 ± 13.790 (62); 58
Menopausal status at diagnosis	Pre-menopause	213 (31.6%)
	Peri-menopause	17 (2.5%)
	Post-menopause	328 (48.7%)
Histological subtype	IDC	533 (79.2%)
	ILC	84 (12.5%)
	Mucinous	8 (1.2%)
	Other	15 (2.2%)
	DCIS	33 (4.9%)
Tumour stage	T0	43 (6.4%)
	T1	257 (38.2%)
	T2	304 (45.2%)
	T3	50 (7.4%)
	T4	8 (1.2%)
Tumour grade	0	1 (0.1%)
	1	59 (8.8%)
	2	390 (57.9%)
	3	167 (24.8%)
N-stage	0	402 (59.7%)
	1	167 (24.8%)
	2	59 (8.8%)
	3	34 (5.1%)
Tumour size (mm)	Mean ± SD (range); median	25.71 ± 16.191 (100); 22
Bilateral cancer	Yes	31 (4.6%)
	No	628 (93.3%)
Lymphatic invasion	Yes	61 (9.1%)
	No	152 (22.6%)
Associated DCIS	Yes	189 (28.1%)
	No	54 (8.0%)
Adjuvant chemotherapy	Yes	289 (42.9%)
	No	371 (55.1%)
Adjuvant radiation therapy	Yes	471 (70%)
	No	186 (27.6%)
Adjuvant endocrine therapy	Yes	510 (75.8%)
	No	158 (23.5%)
Extended endocrine therapy	Yes	80 (11.9%)
	No	239 (35.5%)
ER status	Positive	516 (76.7%)
	Negative	143 (21.2%)
PR status	Positive	432 (64.2%)
	Negative	227 (33.7%)
HER2 status	Positive	106 (15.8%)
	Negative	553 (82.2%)
Recurrence	Yes	121 (18%)
	No	552 (82%)
Local recurrence	Yes	50 (7.4%)
	No	622 (92.4%)
Local recurrence at 5 years	Yes	47 (7.0%)
	No	626 (93%)
Procedure	WLE	345 (51.3%)
	Mastectomy	326 (48.4%)
Axillary procedure	SLNB/SLND	427 (63.4%)
	ALNB/ALND	235 (34.9%)
Metastasis	Yes	91 (13.5%)
	No	581 (86.3%)
Metastasis at 5 years	Yes	69 (10.3%)
	No	604 (89.7%)
Site of metastasis	Bone	35 (5.2%)
	Brain	4 (0.6%)
	Contralateral breast	2 (0.3%)
	En Cuirasse	1 (0.1%)
	Liver	6 (0.9%)
	Lung	11 (1.6%)
	Lymph nodes	8 (1.2%)
	Omentum	1 (0.1%)
	Skin	1 (0.1%)
	Uterus	1 (0.1%)
	Multiple sites	19 (2.8%)
Follow up time (months)	Mean ± SD (range); median	66.85 ± 27.983 (0–140); 65.0
Time to death (years)	Mean ± SD (range); median	3.61 ± 2.374 (0–10); 3.0

*SD* standard deviation, *IDC* invasive ductal carcinoma, *ILC* invasive lobular carcinoma, *DCIS* ductal carcinoma in situ, *ER* oestrogen, *PR* progesterone, *HER2* human epidermal growth factor receptor-2, *WLE* wide local excision, *SLNB* sentinel lymph node biopsy, *SLND* sentinel lymph node dissection, *ALNB* axillary lymph node biopsy, *ALND* axillary lymph node dissection

The mean pre-operative NLR value of patients included in this study was 2.98 ± 1.46 (range 0.07–13.49) which is shown in Table 2.

**Table 2:** Neutrophil–lymphocyte ratio

	Mean	Median	SD	Range
NLR	2.98	2.64	1.46	13.42

### Correlation between neutrophil–lymphocyte ratio and clinicopathological data

There was a positive correlation between the age at the time of surgery and the pre-operative NLR (r = 0.086) which was statistically significant (p = 0.027). The tumour size was also positively correlated with the pre-operative NLR (r = 0.164) which was statistically significant (p = 0.001). The mean difference in pre-operative NLR (0.370) among HER2 + and HER2− groups was statistically significant (p = 0.017). Menopause, tumour stage and grade, nodal involvement, histological subtype of the tumour, accompanying DCIS, lymphovascular invasion (LVI), ER/PR status, bilateral presence of BC, neoadjuvant chemotherapy, and the type of procedure were not associated with pre-operative NLR value. The relationship between clinicopathological parameters of breast cancer and serum levels of pre-operative NLR are shown in Table 3.

**Table 3:** Association between clinicopathological characteristics and NLR

	Correlation Test	
Clinicopathological characteristics	Pre-operative NLR	
Age at the time of the surgery	Pearson Correlation	0.086
	p-value (2-tailed)	0.027
Tumour size	Pearson Correlation	0.164
	p-value (2-tailed)	0.001

		ANOVA			
Clinicopathological characteristics	Sum of squares	df	Mean Square	F	p-value
Menopause	434.117	467	0.930	1.005	0.503
pT	315.487	536	0.589	0.914	0.749
pN	388.627	535	0.726	0.960	0.626
Grade	179.632	507	0.354	1.206	0.118
Histological subtype	526.887	543	0.970	0.981	0.567

Clinicopathological characteristics		Independent Samples Test			
		Mean Difference	95% CI		p-value
			Lower	Upper	
Accompanying histology	DCIS	0.157	− 0.218	0.532	0.410
	No DCIS				
LVI	Yes	0.065	− 0.305	0.436	0.729
	No				
ER	ER +	0.069	− 0.203	0.341	0.618
	ER−				
PR	PR +	0.155	− 0.081	0.390	0.198
	PR−				
HER2	HER2 +	0.370	0.065	0.675	**0.017**
	HER2−				
Bilateral cancer	Yes	− 0.363	− 0.892	0.166	0.178
	No				
Procedure	WLE	− 0.161	− 0.383	0.061	0.154
	Mastectomy				

*LVI* lymphovascular invasion

### Regression analyses for neutrophil–lymphocyte ratio and clinicopathological data

Univariable linear regression analysis showed that only age at the time of surgery (β = 0.009, 95% CI 0.001–0.017, P = 0.027), tumour size (β = 0.013, 95% CI 0.005–0.021, P = 0.001), and HER2 status (β = − 0.370, 95% CI − 0.676—−0.065, P = 0.017) were associated with pre-operative NLR. Multivariable linear regression analysis revealed that the tumour size (β = 0.011, 95% CI 0.002–0.019, P 0.013) was the only clinicopathological parameter correlating statistically significantly with pre-operative NLR in this study. The results of the univariable and multivariable linear regression analysis are detailed in Table 4.

**Table 4:** Linear regression analysis of clinicopathological characteristics and pre-operative NLR

	Univariable		Multivariable	
Variables	β (95% CI)	p value	β (95% CI)	p value
Age	0.009 (0.001–0.017)	**0.027**	0.007 (− 0.003–0.017)	0.162
Menopause	0.037 (− 0.090–0.165)	0.564		
T-stage	0.034 (− 0.111–0.178)	0.649		
N-stage	− 0.031 (− 0.161–0.100)	0.647		
Histological subtype	− 0.067 (− 0.180–0.045)	0.238		
Grade	0.063 (− 0.138–0.263)	0.539		
Accompanying histology	− 0.157 (− 0.533–0.218)	0.410		
LVI	− 0.065 (− 0.436–0.306)	0.729		
Tumour size	0.013 (0.005–0.021)	**0.001**	0.011 (0.002–0.019)	**0.013**
ER	− 0.069 (− 0.341–0.203)	0.618		
PR	− 0.155 (− 0.391–0.081)	0.198		
HER2	− 0.370 (− 0.676–− 0.065)	**0.017**	− 0.218 (− 0.612–0.176)	0.277
Bilateral cancer	0.363 (− 0.166–0.892)	0.178		

Statistically significant *p*-values are highlighted in bold

## Discussion

This study has evaluated the predictive value of the pre-operative neutrophil**–**lymphocyte ratio in relation to clinicopathological parameters of BC, such as age, histological subtype, tumour grade, pT stage, pN stage, LVI, tumour size, HER2/ER/PR, and menopausal status. Given the propensity for HER2 tumours to be traditionally considered of aggressive tumour biology [4], it is somewhat unsurprising that our data demonstrated a correlation between HER2 status and NLR. Moreover, aggressive tumour biology tends to be diagnosed at a later stage [23], which supports the notion that increased NLR should be associated with increased tumour burden.

Although there is a growing body of knowledge on this topic, the findings are inconsistent with existing literature, demonstrating the novelty of these results, and also the requirement for more robust analyses of NLR in the clinical setting.

A significant association between age, HER2 status, tumour size, and NLR was observed in univariable analysis. These results demonstrated that preoperative NLR may have a potential predictive value of age, tumour size, and HER2 status in patients with primary BC.

Contrary to this study, Zhu et al. previously reported that NLR was significantly higher in younger and pre-menopausal women, refuting the results of the current study [24].

Concordant with the current study, Jadoon et al. demonstrated that NLR was significantly correlated with tumour size while no difference in histological grading, metastasis, surgical modality, sentinel, or axillary node status was observed [25]. However, they also illustrate NLR to be associated with tumour stage 1 and nodal stage 2/3 which is not consistent with the results of the present study. Moreover, Yang et al. reported that NLR had no significant association with age, tumour size, ER/PR/HER2 status, and Ki67 expression assays, however, did correlate with p53 expression and lymph node metastasis [26]. Sun et al. also described no correlation between NLR and clinicopathological parameters including age, HER2 status, and tumour size [27]. However, this study failed to observe a significant association between age, HER2 status, and NLR in multivariable analysis. This can be attributed to the combined effects of several factors, such as confounding variables (e.g. hormone receptor status, age, tumour size), small sample size, and tumour microenvironment. Supporting these findings, a meta-analysis showed that ER + and HER2 + status weakens the prognostic value of NLR in disease-free survival [28]. In light of this finding, a small percentage (15.8%) of HER2 + patients in this cohort could have contributed to the significant univariate association, while a large percentage (76.7%) of ER + patients and perhaps differences in HER2 + vs HER2− tumour microenvironments could contribute the insignificant multivariate association between HER2 status and NLR. Further studies should explore a multi-biomarker approach to enhance predictive accuracy. While these results add fuel to this clinical conundrum, it is of importance to note that the current data was derived from a significantly larger database from a high-volume tertiary referral centre retrospectively for the treatment of patients diagnosed with primary breast cancer.

These results also demonstrated a significant relationship between NLR and tumour size, thus, suggesting that increased acute phase reactants may be expected with increasing tumour burden. Interestingly, Takeuchi et al. also reported a significant relationship between NLR and tumour size in their previous analysis [29], supporting these findings. These findings have potential applications for predicting drug efficacy and tumour recurrence risk in future studies.

There are limitations to this study. First, this study is of retrospective design rendering it likely to be subject to selection, confounding, and ascertainment biases. Second, this is a single-centre study that did not use an external validation cohort, which would prove fruitful in further ascertaining the relevance of these findings in clinical practice. Additionally, this study excluded patients who had metastatic disease or received neoadjuvant chemotherapy. Studying NLR in these patient groups would be important for future studies. Moreover, heterogeneity in treatment plans, although reflecting real clinical practice, is potentially a confounding factor. Another limitation of this study is not taking all inflammatory factors into account, such as smoking, comorbidities, medications, and patients who received core biopsy versus who did not.While these results are of interest for translational research purposes, this study fails to cast light on the relevance of such results in clinical practice, as NLR will not deter formal staging and histopathological tumour evaluation through the multidisciplinary process. Finally, our scope was limited to the association between NLR and clinicopathological characteristics. Comparing pre- and post-operative NLR could be of additional value in post-operative disease surveillance and prognosis, hence, should also be considered in future studies.

In conclusion, this study demonstrates that pre-operative NLR has an independent predictive value in terms of tumour size in patients being treated with primary BC. Ratification of these preliminary findings is warranted before robustly adopted into clinical practice.

## Data Availability

Data is provided within the manuscript.
