# Correction: Assessing the clinical utility of pre-operative neutrophil–lymphocyte ratio as a predictor of clinicopathological parameters in patients being treated for primary breast cancer

**DOI:** 10.1007/s10549-025-07698-3

**Published:** 2025-04-18

**Authors:** Burce Isik, Matthew G. Davey, Alisha A. Jaffer, Juliette Buckley, James A. L. Brown, Chwanrow Baban, Bridget Anne Merrigan, Shona Tormey

**Affiliations:** 1 University of Limerick School of Medicine, Limerick, Ireland; 2 Symptomatic Breast Unit, University Hospital Limerick, Limerick, Ireland; 3 Department of Biological Sciences, University of Limerick, Galway, Ireland; 4 Limerick Digital Cancer Research Centre (LDCRC), University of Limerick, Limerick, Ireland

**Correction to: Breast Cancer Research and Treatment (2025) 210:783–790** 10.1007/s10549-025-07615-8

In this article, James A.L. Brown at affiliations ‘Department of Biological Sciences, University of Limerick, Galway, Ireland’ and ‘Limerick Digital Cancer Research Centre (LDCRC), University of Limerick, Limerick, Ireland’ was missing from the author list. The original article has been corrected.

